# Morphological and molecular identification of metacestodes infecting teleost fishes of Moreton Bay, Australia

**DOI:** 10.1007/s11230-024-10183-y

**Published:** 2024-08-21

**Authors:** Scott C. Cutmore, Michael B. Bennett, Thomas H. Cribb

**Affiliations:** 1 Queensland Museum, Biodiversity and Geosciences Program, South Brisbane, QLD 4101 Australia; 2 School of The Environment, The University of Queensland, St Lucia, QLD 4072 Australia; 3 School of Biomedical Sciences, The University of Queensland, St Lucia, QLD 4072 Australia

## Abstract

In a parasitological survey of fishes from Moreton Bay (southeastern Queensland, Australia), 169 teleost fishes, representing 54 species from 28 families, were examined for larval cestodes. Of these 54 species, 36 were found to be infected by metacestodes. Metacestodes were characterised by morphological and molecular data (the D1-D3 region of the 28S rDNA gene); these data were analysed in parallel to inform larval type allocation. Metacestodes collected represented eight morphological types, seven previously reported (Types I, II, IV, V, VI, VII, and X) and one novel type (Type XVI). Phylogenetic analyses were conducted to genetically match larval types to adult cestodes. Six of the eight larval types found were matched to adult forms: Type I metacestodes matched species of *Phoreiobothrium* Linton, 1889 (Onchobothriidae); Type II metacestodes matched species of *Acanthobothrium* van Beneden, 1849 (Onchobothriidae); Type IV metacestodes matched species of *Scyphophyllidium* Woodland, 1927 and *Alexandercestus* Ruhnke & Workman, 2013 (Phyllobothriidae); Type VI metacestodes matched species of *Anthobothrium* van Beneden, 1850 (Tetraphyllidea *incertae sedis*); Type X metacestodes matched species of *Ambitalveolus* Caira & Jensen, 2022 (Tetraphyllidea *incertae sedis*); and Type XVI metacestodes matched species of *Platybothrium* Linton, 1890 (Onchobothriidae). Based on phylogenetic topology, Type V metacestodes are inferred to match *Pedibothrium* Linton, 1909 (Balanobothriidae) and Type VII metacestodes are inferred to match *Spongiobothrium* Linton, 1889 (Rhinebothriidae). These findings support and extend the unified morphological type system proposed previously, but suggest that morphological types will ultimately be informative to identify metacestodes to a group of related genera rather than any distinct genus.

## Introduction

Since the original descriptions of *Scolex pleuronectis* Müller, 1788 and *Scolex polymorphus* Rudolphi, 1819, there has been disorder in the identification of “tetraphyllidean” metacestodes. The lack of development in scolex morphology at this stage, and the usually complete absence of strobila development, has rendered metacestodes notoriously challenging to identify. While identification has occasionally been attempted using morphological techniques (e.g., Cake, 1976; Carvajal et al., 1982; Chambers et al., 2000; Hamilton & Byram, 1974), more often than not, identification to species or genus was not attempted at all and specimens have simply been reported as metacestode morphotypes. Chambers et al. (2000) provided the only comprehensive Australian report of metacestodes infecting teleosts, describing 11 metacestode types, from teleosts off Heron Island on the Great Barrier Reef. These authors assigned putative generic identities to the metacestode types using morphological features and, in some cases, on the basis of some *in vitro* development (Chambers et al., 2000).

Over the last two decades, however, there have been substantial advances in our understanding of metacestode identities, with molecular data enabling the definitive association of metacestode types to their adult forms (e.g., Agustí et al., 2005; Aznar et al., 2007; Brickle et al., 2001; Gordeev & Sokolov, 2016; Holland & Wilson, 2009; Jensen & Bullard, 2010; Randhawa, 2011; Tedesco et al., 2020). In the most comprehensive review of marine metacestodes to date, Jensen & Bullard (2010) drew on both morphological and molecular data to present a classification of “tetraphyllidean” and rhinebothriidean metacestodes. These authors proposed a classification scheme of 15 larval types (Types I–XV), eight of which were characterised by partial 28S rDNA sequence data. Seven of these eight types were genetically matched to known genera: Larval Type I was linked to *Phoreiobothrium* Linton, 1889 and *Triloculatum* Caira & Jensen, 2009; Type II to *Acanthobothrium* van Beneden, 1849; Type III to *Duplicibothrium* Williams & Campbell, 1978; Type IV to *Paraorygmatobothrium* Ruhnke, 1994 (now *Scyphophyllidium* Woodland, 1927); Type VI to *Anthobothrium* van Beneden, 1850; Type VII to *Rhinebothrium* Linton, 1890 and *Spongiobothrium* Linton, 1889; and Type VIII to *Rhodobothrium* Linton, 1889. Despite the major advances represented by the work of Jensen & Bullard (2010), there is clearly more that remains to be clarified in the identification and biology of these metacestodes.

In this study we integrate morphological and molecular data to expand on the work of Jensen & Bullard (2010). This study focuses on piscivorous elasmobranchs of the orders Carcharhiniformes and Orectolobiformes as definitive hosts and the infraclass Teleostei as intermediate hosts, all within a single locality, Moreton Bay, in southeastern Queensland, Australia. The cestode fauna of Moreton Bay, although incompletely described, is rich and provides an ideal location for a study of this type as several genera absent from the analysis of Jensen & Bullard (2010) have been reported, and genetically characterised, from the region (Cutmore et al., 2010; Cutmore et al., 2017; Cutmore et al., 2018; Cutmore et al., 2011).

## Methods

### Sample collection

Teleost fishes belonging to 29 families (Table 1) were collected from eastern Moreton Bay (27°26'S, 153°24'E) and western Moreton Bay (27°22'S, 153°13'E) by baited lines, seine nets, cast nets or sourced from the commercial fishery. Systematics of host fishes follows that of FishBase (Froese & Pauly, 2023). Intestines were removed, opened longitudinally and examined under a dissecting microscope. Metacestodes found were washed and subsequently killed in near-boiling vertebrate saline (0.85% NaCl solution) and fixed 10% formalin for morphological examination and in 100% ethanol for molecular analysis. Larval cestodes were analysed as paragenophore pairs (hologenophores *sensu* Pleijel et al., 2008), where two morphologically identical specimens were processed for parallel morphological (one specimen) and molecular (one specimen) analyses.

**Table 1:** Teleost species examined for metacestodes during this study. Circles indicate a larval type identification demonstrated by molecular data and triangles indicate an identification demonstrated by only morphology.

Host	# dissected/ infected	Larval Type I	Larval Type II	Larval Type IV	Larval Type V	Larval Type VI	Larval Type VII	Larval Type X	Larval Type XVI	Total # types
**ACANTHURIFORMES**										
**Scatophagidae**										
*Selenotoca multifasciata* (Richardson)	2/0									0
**Siganidae**										
*Siganus fuscescens* (Houttuyn)	4/2			▲		▲				2
**ANGUILLIFORMES**										
**Muraenesocidae**										
*Muraenesox bagio* (Hamilton)	1/0									0
**ATHERINIFORMES**										
**Atherinidae**										
*Atherinomorus vaigiensis* (Quoy & Gaimard)	12/0									0
**AULOPIFORMES**										
**Synodontidae**										
*Saurida undosquamis* (Richardson)	6/6	▲	●	●		●			▲	5
**CARANGIFORMES**										
**Carangidae**										
*Alepes apercna* Grant	2/1					▲				1
*Caranx sexfasciatus* Quoy & Gaimard	1/0									0
**CENTRARCHIFORMES**										
**Kyphosidae**										
*Microcanthus strigatus* (Cuvier)	3/3	●	●	●	▲	●				5
*Scorpis lineolata* Kner	3/3	●			▲	▲				3
**Terapontidae**										
*Pelates quadrilineatus* (Bloch)	1/0									0
**KURTIFORMES**										
**Apogonidae**										
*Ostorhinchus limenus* (Randall & Hoese)	5/5	●	●	●	▲	●				5
**MUGILIFORMES**										
**Mugilidae**										
*Gracilimugil argenteus* (Quoy & Gaimard)	1/1					●				1
*Mugil cephalus* Linnaeus	1/0									0
*Planiliza subviridis* (Valenciennes)	2/0									0
**PERCIFORMES**										
**Pinguipedidae**										
*Parapercis nebulosa* (Quoy & Gaimard)	1/1		●	▲		●			●	4
**Platycephalidae**										
*Platycephalus endrachtensis* Quoy & Gaimard	1/0									0
*Platycephalus fuscus* Cuvier	7/6	▲				▲			▲	3
*Platycephalus australis* Imamura	3/3					●			▲	2
**Serranidae**										
*Epinephelus coioides* (Hamilton)	1/0									0
*Epinephelus undulatostriatus* (Peters)	1/1					●				1
**Uranoscopidae**										
*Ichthyscopus sannio* Whitley	1/0									0
**SCOMBRIFORMES**										
**Pomatomidae**										
*Pomatomus saltatrix* (Linnaeus)	9/3	●				●				2
**Scombridae**										
*Euthynnus affinis* (Cantor)	3/0									0
*Scomberomorus munroi* Collette & Russo	2/0									0
*Scomberomorus queenslandicus* Munro	1/1	●								1
**PLEURONECTIFORMES**										
**Paralichthyidae**										
*Pseudorhombus arsius* (Hamilton)	3/3		●	▲		●	●			4
*Pseudorhombus jenynsii* (Bleeker)	2/2		●	●		●			▲	4
**SILURIFORMES**										
**Ariidae**										
*Neoarius graeffei* (Kner & Steindachner)	1/1			▲		●			▲	3
**TETRAODONTIFORMES**										
**Diodontidae**										
*Tragulichthys jaculiferus* (Cuvier)	1/0									0
**CARANGARIA *INCERTAE SEDIS***										
**Sphyraenidae**										
*Sphyraena obtusata* Cuvier	5/5	●		●		●				3
**EUPERCARIA* INCERTAE SEDIS***										
**Gerreidae**										
*Gerres oyena* (Forsskål)	1/0									0
*Gerres subfasciatus* Cuvier	13/0									0
**Labridae**										
*Choerodon cephalotes* (Castelnau)	1/1			●						1
*Pseudolabrus guentheri* Bleeker	5/4	▲	●			●		●	▲	5
*Thalassoma jansenii* (Bleeker)	1/1	▲	●			▲	●			4
*Thalassoma lunare* (Linnaeus)	4/4		●	●		●		●		4
*Thalassoma lutescens* (Lay & Bennett)	1/1		▲			●				2
**Lethrinidae**										
*Lethrinus genivittatus* Valenciennes	4/4				●	▲				2
*Lethrinus laticaudis* Alleyne & Macleay	2/0									0
**Lutjanidae**										
*Lutjanus fulviflamma* (Forsskål)	2/2	●		▲		▲			▲	4
**Monodactylidae**										
*Monodactylus argenteus* (Linnaeus)	4/4			▲		●				2
**Nemipteridae**										
*Pentapodus paradiseus* (Günther)	7/7	▲	●			●			▲	4
**Sillaginidae**										
*Sillago analis* Whitley	2/0									0
*Sillago ciliata* Cuvier	5/1			▲		▲				2
*Sillago maculata* Quoy & Gaimard	6/5	●				●			●	3
**Sparidae**										
*Acanthopagrus australis* (Günther)	4/1	▲								1
*Chrysophrys auratus* (Forster)	4/4	●		▲		●				3
*Rhabdosargus sarba* (Forsskål)	5/4	●				●			●	3
**OVALENTARIA *INCERTAE SEDIS***										
**Pomacentridae**										
*Abudefduf bengalensis* (Bloch)	3/3	▲			●	●			●	4
*Abudefduf sordidus* (Forsskål)	2/2	●				▲				2
*Abudefduf vaigiensis* (Quoy & Gaimard)	1/1	▲				●				2
*Abudefduf whitleyi* Allen & Robertson	4/4	▲		▲	●	●				4
*Parma oligolepis* Whitley	1/1	▲				▲				2
*Plectroglyphidodon gascoynei* (Whitley)	1/0									0
**Total number of species infected**	**169/101**	**21**	**11**	**16**	**6**	**33**	**2**	**2**	**12**	

Carcharhiniform and orectolobiform sharks (Table 2) were collected from eastern Moreton Bay (27°26'S, 153°24'E), western Moreton Bay (27°22'S, 153°13'E) and the Brisbane River (27°31'S, 152°59'E), Queensland, using gill nets, seine nets and baited lines or sourced from the commercial fishery. All shark hosts were identified to species using Last & Stevens (2009). Specimens of *Chiloscyllium punctatum* Müller & Henle are reported in this study as *Chiloscyllium* cf. *punctatum*, as the status of this shark species in Australian waters remains ambiguous (see Cutmore et al., 2010; Naylor et al., 2012). Sharks were euthanised by neural pithing and spiral intestines were removed, opened longitudinally and examined under a dissecting microscope. Cestodes were removed, washed and subsequently killed in near-boiling saline solution (0.85% NaCl solution). Worms were fixed in 10% formalin for morphological examination and in 100% ethanol for molecular analysis. Some individual worms were fixed for both morphological and molecular analysis (hologenophores *sensu* Pleijel et al., 2008). For these specimens the anterior two-fifths and posterior two-fifths of the worm were fixed in 10% formalin and the middle fifth in 100% ethanol.

**Table 2:** Carcharhiniform and orectolobiform shark species examined during this study.

**Host species**	**# dissected/ infected**	**Genera found**
**CARCHARHINIFORMES**		
**Carcharhinidae**		
*Carcharhinus amboinensis* (Müller & Henle)	6/5	*Anthobothrium*; *Platybothrium*; *Scyphophyllidium*
*Carcharhinus cautus* (Whitley)	7/7	*Anthobothrium*; *Platybothrium*;* Scyphophyllidium*
*Carcharhinus leucas* (Müller & Henle)	6/6	*Alexandercestus*; *Anthobothrium*; *Phoreiobothrium*; *Platybothrium*;* Scyphophyllidium*
*Carcharhinus limbatus* (Müller & Henle)	12/12	*Anthobothrium*; *Phoreiobothrium*; *Platybothrium*;* Scyphophyllidium*
*Carcharhinus obscurus* (Lesueur)	4/3	*Anthobothrium*; *Platybothrium*;* Scyphophyllidium*
*Carcharhinus sorrah* (Müller & Henle)	4/4	*Anthobothrium*; *Platybothrium*;* Scyphophyllidium*
*Galeocerdo cuvier* (Péron & Lesueur)	1/1	*Scyphophyllidium*;* Thysanocephalum*
*Rhizoprionodon acutus* (Rüppell)	1/0	
*Rhizoprionodon taylori* (Ogilby)	19/0	
**Hemigaleidae**		
*Hemigaleus australiensis* White, Last & Compagno	9/7	*Scyphophyllidium*
*Hemipristis elongata* (Klunzinger)	2/2	*Hemipristicola*; *Megalonchos*;* Scyphophyllidium*
**Sphyrnidae**		
*Sphyrna lewini* (Griffith & Smith)	8/8	*Phoreiobothrium*;* Scyphophyllidium*
*Sphyrna mokarran* (Rüppell)	3/3	*Platybothrium*;* Scyphophyllidium*
**ORECTOLOBIFORMES**		
**Hemiscylliidae**		
*Chiloscyllium* cf. *punctatum* Müller & Henle	7/7	*Caulopatera*; *Scyphophyllidium*; *Spiniloculus*;* Yorkeria*
**Orectolobidae**		
*Orectolobus maculatus* (Bonnaterre)	8/8	*Acanthobothrium*;* Scyphophyllidium*
*Orectolobus ornatus* (De Vis)	6/6	*Acanthobothrium*; *Ambitalveolus*;* Scyphophyllidium*
**Total**	**103/79**	

### Morphological analysis

Specimens for morphological analysis were washed in fresh water, stained in Mayer's haematoxylin, destained in a solution of 1.0% hydrochloric acid, and neutralised in 1.0% ammonium hydroxide solution. Specimens were then dehydrated through a graded ethanol series, cleared in methyl salicylate, and mounted in Canada balsam. Measurements were made using an Olympus SC50 digital camera mounted on an Olympus BX-53 compound microscope using cellSens Standard imaging software. Measurements are in micrometres unless otherwise stated and are given as the range followed by the mean in parentheses. Drawings were made using an Olympus BX-53 compound microscope and drawing tube. Voucher specimens are lodged in the Queensland Museum (QM), Brisbane, Australia.

### Molecular and phylogenetic analysis

Specimens for molecular analyses were processed according to the protocols used by Cutmore et al. (2017), with total genomic DNA extracted using phenol/chloroform extraction techniques (Sambrook & Russell, 2001) and the partial D1-D3 region of the large (28S) ribosomal subunit RNA coding region amplified and sequenced using the primers LSU5 (5'-TAG GTC GAC CCG CTG AAY TTA AGC-3'; Littlewood, 1994), 300F (5'-CAA GTA CCG TGA GGG AAA GTT-3'; Littlewood et al., 2000), ECD2 (5'-CTT GGT CCG TGT TTC AAG ACG GG-3'; Littlewood et al., 1997), and 1200R (5'-GCA TAG TTC ACC ATC TTT CGG-3'; Lockyer et al., 2003). Geneious® version 10.2.3 (Kearse et al., 2012) was used to assemble and edit contiguous sequences.

The partial 28S rDNA data generated during this study were aligned with relevant sequence data available on GenBank (Tables 3 and 4) using MUSCLE version 3.7 (Edgar, 2004) run on the CIPRES portal (Miller et al., 2010), with ClustalW sequence weighting and UPGMB clustering for iterations 1 and 2. The resultant alignments were refined by eye using Maddison & Maddison (2024); the ends of the alignments were trimmed, and indels constituting more than three base positions and present in greater than 5% of the sequences in the dataset were removed. Maximum likelihood analyses were conducted to explore relationships among cestode taxa, using RAxML version 8.2.12 (Stamatakis, 2014), run on the CIPRES portal. Nodal support in the maximum likelihood analyses was estimated by performing 1,000 bootstrap pseudoreplicates. Functional outgroup taxa for each analysis were chosen based on the most recent phylogenetic topologies for the relevant group.

**Table 3:** Sequences of metacestodes from GenBank included in this study.

**Species**	**Host species**	**GenBank accession #**	**Reference**
Type I MS05-101-10	*Lobotes surinamensis*	GQ470059	Jensen & Bullard (2010)
Type I MS05-292-9	*Opsanus beta*	GQ470062	Jensen & Bullard (2010)
Type I MS05-34-19	*Trichiurus lepturus*	GQ470085	Jensen & Bullard (2010)
Type I MS05-36-14	*Trichiurus lepturus*	GQ470099	Jensen & Bullard (2010)
Type II MS05-218-1	*Lagodon rhomboides*	GQ470115	Jensen & Bullard (2010)
Type II MS05-47-3	*Diplectrum formosum*	GQ470116	Jensen & Bullard (2010)
Type II MS05-47-16	*Diplectrum formosum*	GQ470120	Jensen & Bullard (2010)
Type II 1-37	*Trachinotus rhodopus*	MZ048232	Adán-Torres et al. (2022)
Type II 16/04	*Octopus vulgaris*	MN660287	Tedesco et al. (2020)
Type II 16/30	*Octopus vulgaris*	MN660288	Tedesco et al. (2020)
Type IV MS05-272-14	*Paralichthys lethostigma*	GQ470008	Jensen & Bullard (2010)
Type IV MS05-34-15	*Trichiurus lepturus*	GQ470022	Jensen & Bullard (2010)
Type IV MS05-292-8	*Opsanus beta*	GQ470030	Jensen & Bullard (2010)
Type IV MS05-568-3	*Cynoscion nebulosus*	GQ470050	Jensen & Bullard (2010)
Type V MS05-25-1	*Lutjanus campechanus*	GQ470153	Jensen & Bullard (2010)
Type V MS05-292-10	*Opsanus beta*	GQ470155	Jensen & Bullard (2010)
Type VI MS05-34-20	*Trichiurus lepturus*	GQ470158	Jensen & Bullard (2010)
Type VI MS05-34-21	*Trichiurus lepturus*	GQ470162	Jensen & Bullard (2010)
Type VI MS05-34-17	*Trichiurus lepturus*	GQ470167	Jensen & Bullard (2010)
Type VI MN2022	*Sepioteuthis lessoniana*	LC771487	Nitta et al. (2023)
Type VI 16/06b	*Octopus vulgaris*	MN660290	Tedesco et al. (2020)
Type VII MS05-40-1	*Stenotomus caprinus*	GQ470194	Jensen & Bullard (2010)
Type VII MS05-137-1	*Donax variabilis*	GQ470175	Jensen & Bullard (2010)
Type VII MS05-176-7	*Urophycis floridana*	GQ470183	Jensen & Bullard (2010)

**Table 4:** Sequences of adult cestodes from GenBank included in this study.

**Species**	**Host species**	**GenBank accession #**	**Reference**
**ONCHOPROTEOCEPHALIDEA**			
**Onchobothriidae**			
*Acanthobothrium cleofanus*	*Hypanus longus*	MZ081426	Adán-Torres et al. (2022)
*Acanthobothrium hypermekkolpos*	*Rhynchobatus laevis*	HQ917929	Fyler & Caira (2010)
*Acanthobothrium jeanneae*	*Rhynchobatus laevis*	HQ917928	Fyler & Caira (2010)
*Acanthobothrium katherineae*	*Squaliolus aliae*	MT395344	Gallagher & Caira (2020)
*Acanthobothrium margieae*	*Orectolobus maculatus*	MH729997	Cutmore et al. (2018)
*Acanthobothrium masnihae*	*Urogymnus polylepis*	FJ843604	Fyler & Caira (2010)
*Acanthobothrium matttaylori*	*Rhynchobatus laevis*	HQ917927	Fyler & Caira (2010)
*Acanthobothrium popi*	*Himantura* sp.	FJ843600	Fyler et al. (2009)
*Acanthobothrium romanowi*	*Himantura* sp.	FJ843598	Fyler et al. (2009)
*Acanthobothrium zimmeri*	*Himantura* sp.	FJ843602	Fyler et al. (2009)
*Acanthobothrium* sp.	*Himantura* sp.	FJ843593	Fyler et al. (2009)
*Phoreiobothrium lewinense*	*Sphyrna lewini*	KF685896	Caira et al. (2014)
*Phoreiobothrium* sp. 1A	*Carcharhinus brevipinna*	GQ470064	Jensen & Bullard (2010)
*Platybothrium auriculatum*	*Prionace glauca*	KF685898	Caira et al. (2014)
*Platybothrium jondoeorum*	*Negaprion acutidens*	KF685772	Caira et al. (2014)
*Triloculatum andersonorum*	*Negaprion acutidens*	KF685895	Caira et al. (2014)
*Triloculatum bullardi*	*Carcharhinus brevipinna*	GQ470102	Jensen & Bullard (2010)
*Megalonchos shawae*	*Hemipristis elongata*	MH729992	Cutmore et al. (2018)
*Megalonchos sumansinghai*	*Hemipristis elongata*	MH729993	Cutmore et al. (2018)
**Proteocephalidae**			
*Gangesia parasiluri*	*Silurus asotus*	AF286935	Olson et al. (2001)
*Peltidocotyle rugosa*	*Pseudoplatystoma fasciatum*	AF286937	Olson et al. (2001)
*Proteocephalus macrocephalus*	*Anguilla anguilla*	EF095261	Waeschenbach et al. (2007)
***Incertae sedis***			
*Matticestus anneae*	*Pristis clavata*	MG952948	Caira et al. (2018)
*Matticestus kathleenae*	*Pristis clavata*	MG952946	Caira et al. (2018)
**PHYLLOBOTHRIIDEA**			
**Phyllobothriidae**			
*Alexandercestus gibsoni*	*Carcharhinus leucas*	MG008925	Cutmore et al. (2017)
*Clistobothrium amyae*	*Pseudocarcharias kamoharai*	MN706184	Caira et al. (2020)
*Clistobothrium gabywalterorum*	*Pseudocarcharias kamoharai*	MN706183	Caira et al. (2020)
*Hemipristicola gunterae*	*Hemipristis elongata*	HQ680623	Cutmore et al. (2011)
*Scyphophyllidium bai*	*Mustelus mustelus*	KC505625	Ruhnke & Workman (2013)
*Scyphophyllidium christopheri*	*Carcharhinus sorrah*	MG008931	Cutmore et al. (2017)
*Scyphophyllidium exiguum*	*Alopias vulpinus*	KF685769	Caira et al. (2014)
*Scyphophyllidium guariticus*	*Paratrygon aiereba*	KF685888	Caira et al. (2014)
*Scyphophyllidium harti*	*Carcharhinus leucas*	MG008939	Cutmore et al. (2017)
*Scyphophyllidium janineae*	*Hemipristis elongata*	HQ680625	Cutmore et al. (2011)
*Scyphophyllidium latipi*	*Scoliodon macrorhynchos*	KF685900	Caira et al. (2014)
*Scyphophyllidium orectolobi*	*Orectolobus maculatus*	MG008940	Cutmore et al. (2017)
*Scyphophyllidium paulum*	*Galeocerdo cuvier*	HQ680628	Cutmore et al. (2011)
*Scyphophyllidium prionacis*	*Prionace glauca*	KF685892	Caira et al. (2014)
*Scyphophyllidium randyi*	*Chiloscyllium hasseltii*	KF685767	Caira et al. (2014)
*Scyphophyllidium sinclairtaylori*	*Carcharhinus sorrah*	MG008933	Cutmore et al. (2017)
*Scyphophyllidium taylori*	*Hemigaleus australiensis*	HQ680631	Cutmore et al. (2011)
*Scyphophyllidium timvickiorum*	*Pseudocarcharias kamoharai*	MN706182	Caira et al. (2020)
*Scyphophyllidium tyleri*	*Chiloscyllium* cf.* punctatum*	MG008930	Cutmore et al. (2017)
*Scyphophyllidium ullmanni*	*Carcharhinus cautus*	MG008942	Cutmore et al. (2017)
*Scyphophyllidium* sp. 2	*Rhizoprionodon terraenovae*	GQ470021	Jensen & Bullard (2010)
*Scyphophyllidium* sp. 3B	*Carcharhinus limbatus*	GQ470005	Jensen & Bullard (2010)
*Scyphophyllidium* sp. 5A	*Carcharhinus brevipinna*	GQ470031	Jensen & Bullard (2010)
*Scyphophyllidium* sp. 6	*Carcharhinus brevipinna*	GQ470001	Jensen & Bullard (2010)
*Scyphophyllidium* sp. Sl168A	*Sphyrna lewini*	MG008929	Cutmore et al. (2017)
*Thysanocephalum thysanocephalum*	*Galeocerdo cuvier*	MG008946	Cutmore et al. (2017)
**RHINEBOTHRIIDEA**			
**Echeneibothriidae**			
*Echeneibothrium megalosoma*	*Dipturus chilensis*	KY569550	Bueno & Caira (2017)
*Echeneibothrium multiloculatum*	*Dipturus chilensis*	KY569546	Bueno & Caira (2017)
**Rhinebothriidae**			
*Rhinebothrium flexile*	*Bathytoshia centroura*	OQ429321	Herzog et al. (2023)
*Rhinebothrium megacanthophallus*	*Urogymnus polylepis*	FJ177120	Healy et al. (2009)
*Rhinebothrium* cf.* oligotesticulare*	*Glaucostegus granulatus*	MT032161	Golzarianpour et al. (2020)
*Rhinebothrium* sp. 4	*Hemitrygon akajei*	FJ177126	Healy et al. (2009)
*Rhinebothrium* sp. 5	*Dasyatis brevis*	FJ177127	Healy et al. (2009)
*Rhinebothroides* cf. *freitasi*	*Potamotrygon falkneri*	FJ177132	Healy et al. (2009)
*Rhabdotobothrium anterophallum*	*Mobula hypostoma*	GQ470179	Jensen & Bullard (2010)
*Rhodobothrium paucitesticulare*	*Rhinoptera bonasus*	GQ470172	Jensen & Bullard (2010)
*Scalithrium* sp.	*Hypanus longus*	KF685878	Caira et al. (2014)
*Spongiobothrium* sp.	*Dasyatis sabina*	GQ470184	Jensen & Bullard (2010)
*Spongiobothrium* sp.	*Rhynchobatus* cf.* australiae*	FJ177134	Healy et al. (2009)
*Spongiobothrium* sp.	*Dasyatis brevis*	AF382085	Brickle et al. (2001)
**TETRAPHYLLIDEA**			
**Balanobothriidae**			
*Balanobothrium* sp.	*Stegostoma tigrinum*	KF685880	Caira et al. (2014)
*Pachybothrium hutsoni*	*Nebrius ferrugineus*	EF095260	Waeschenbach et al. (2007)
*Pedibothrium mounseyi*	*Nebrius ferrugineus*	KF685893	Caira et al. (2014)
*Pedibothrium veravalensis*	*Stegostoma tigrinum*	KF685894	Caira et al. (2014)
*Spiniloculus mavensis*	*Chiloscyllium* cf.* punctatum*	MH729994	Cutmore et al. (2018)
*Spiniloculus* sp.	*Chiloscyllium punctatum*	KF685775	Caira et al. (2014)
*Yorkeria hilli*	*Chiloscyllium punctatum*	KF685903	Caira et al. (2014)
*Yorkeria izardi*	*Chiloscyllium* cf.* punctatum*	KF685904	Caira et al. (2014)
*Yorkeria williamsi*	*Chiloscyllium* cf.* punctatum*	MH729995	Cutmore et al. (2018)
**Gastrolecithidae**			
*Ceratobothrium xanthocephalum*	*Isurus oxyrinchus*	KF685756	Caira et al. (2014)
**Serendipeidae**			
*Duplicibothrium jeannettae*	*Rhinoptera marginata*	OK358920	Stephan & Caira (2022)
*Duplicibothrium minutum*	*Rhinoptera bonasus*	OK358918	Stephan & Caira (2022)
***Incertae sedis***			
*Ambitalveolus penghuensis*	*Orectolobus japonicus*	OM213001	Caira & Jensen (2022)
*Anthobothrium caseyi*	*Prionace glauca*	KF685879	Caira et al. (2014)
*Anthobothrium* sp. 1	*Carcharhinus tilstoni*	KF685752	Caira et al. (2014)
*Anthobothrium* sp. 1A	*Carcharhinus isodon*	GQ470169	Jensen & Bullard (2010)
*Anthobothrium* sp. 1C	*Rhizoprionodon terraenovae*	GQ470161	Jensen & Bullard (2010)
*Anthobothrium* sp. 2A	*Carcharhinus isodon*	GQ470160	Jensen & Bullard (2010)
*Carpobothrium chiloscylli*	*Chiloscyllium indicum*	OM213007	Caira & Jensen (2022)
*Carpobothrium eleanorae*	*Chiloscyllium hasseltii*	OM213009	Caira & Jensen (2022)
*Carpobothrium marjorieae*	*Chiloscyllium* sp.	OM213005	Caira & Jensen (2022)
*Carpobothrium megaphallum*	*Chiloscyllium griseum*	OM213004	Caira & Jensen (2022)
*Caulobothrium opisthorchis*	*Myliobatis californica*	FJ177106	Healy et al. (2009)
*Caulopatera pagei*	*Chiloscyllium* cf.* punctatum*	OM213003	Caira & Jensen (2022)

## Results

### Metacestodes

**Fig. 1:**
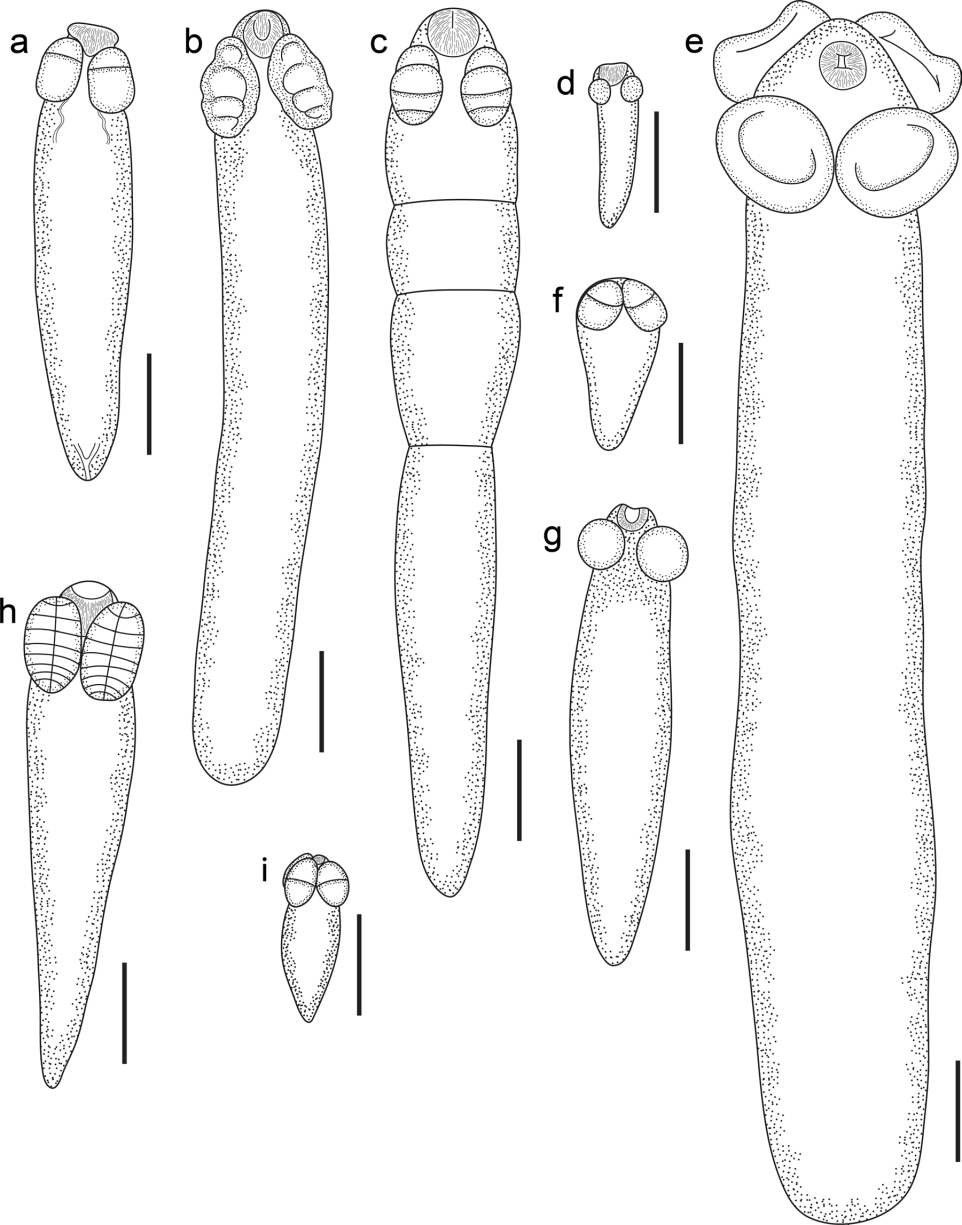
Metacestodes types infecting teleost fishes of Moreton Bay, drawn to scale. **a**, Larval Type I. **b**, Larval Type II, unsegmented. **c**, Larval Type II, segmented. **d**, Larval Type IV. **e**, Larval Type X. **f**, Larval Type V. **g**, Larval Type VI. **h**, Larval Type VII. **i**, Larval Type XVI. Scale-bars: 200 µm.

A total of 169 individual teleosts, representing 54 species from 29 families, were examined for metacestodes. All larval cestodes were collected from the intestine and pyloric caeca. Of the 169 individual teleosts examined, 101 were infected (Table 1). The metacestodes collected represent eight morphological types (Fig. 1), seven corresponding to those of Jensen & Bullard (2010) (Types I, II, IV, V, VI, VII, and X) and one new type, which we describe as Type XVI. Each of the metacestode types found in this study are reported below, with images and measurements provided for the new material. Morphological vouchers and genetic data for metacestode specimens are lodged in the Queensland Museum (G233050–186) and in GenBank (PQ146203–226), respectively.

### Adult cestodes

Sixteen species of carcharhiniform and orectolobiform sharks, representing five families and eight genera, were examined for the presence of adult cestodes. Of the 103 individuals examined, 79 were infected with adult cestodes (Table 2), belonging to 13 genera: *Alexandercestus* Ruhnke & Workman, 2013, *Acanthobothrium*, *Ambitalveolus* Caira & Jensen, 2022, *Anthobothrium*, *Caulopatera* Cutmore, Bennett & Cribb, 2010, *Hemipristicola* Cutmore, Theiss, Bennett & Cribb, 2011, *Megalonchos* Baer & Euzet, 1962, *Phoreiobothrium*, *Platybothrium*, *Scyphophyllidium*, *Spiniloculus* Southwell, 1925, *Thysanocephalum* and *Yorkeria* Southwell, 1927. Morphological and molecular identification of adult worms belonging to *Alexandercestus*, *Phoreiobothrium*, *Scyphophyllidium*, and *Thysanocephalum* were reported in Cutmore et al. (2017), *Caulopatera* in Cutmore et al. (2010) and Caira & Jensen (2022), *Hemipristicola* in Cutmore et al. (2011), and *Acanthobothrium*, *Megalonchos*, *Spiniloculus*, and *Yorkeria* in Cutmore et al. (2018). Specimens of the remaining genera (*Ambitalveolus*, *Anthobothrium*, *Phoreiobothrium*, and *Platybothrium*) were not identified to species, and are reported only to genus; morphological vouchers and genetic data for these four genera are lodged in the Queensland Museum (G232778–855, G232871–936, and G233024) and in GenBank (PQ146187–202), respectively.

### Phylogenetic results

The partial 28S rDNA sequences generated for adult and larval cestodes were initially analysed with all available cestode taxa on GenBank (phylogenetic tree not shown). Metacestode specimens matched adult specimens in several unrelated clades across the Eucestoda phylogenetic tree. Incorporation of the massive amount of data across the entire Eucestoda resulted in substantial alignment-induced data loss. Thus, to increase species-level resolution, the six clades with matching metacestode data were each aligned and analysed separately.

**Fig. 2:**
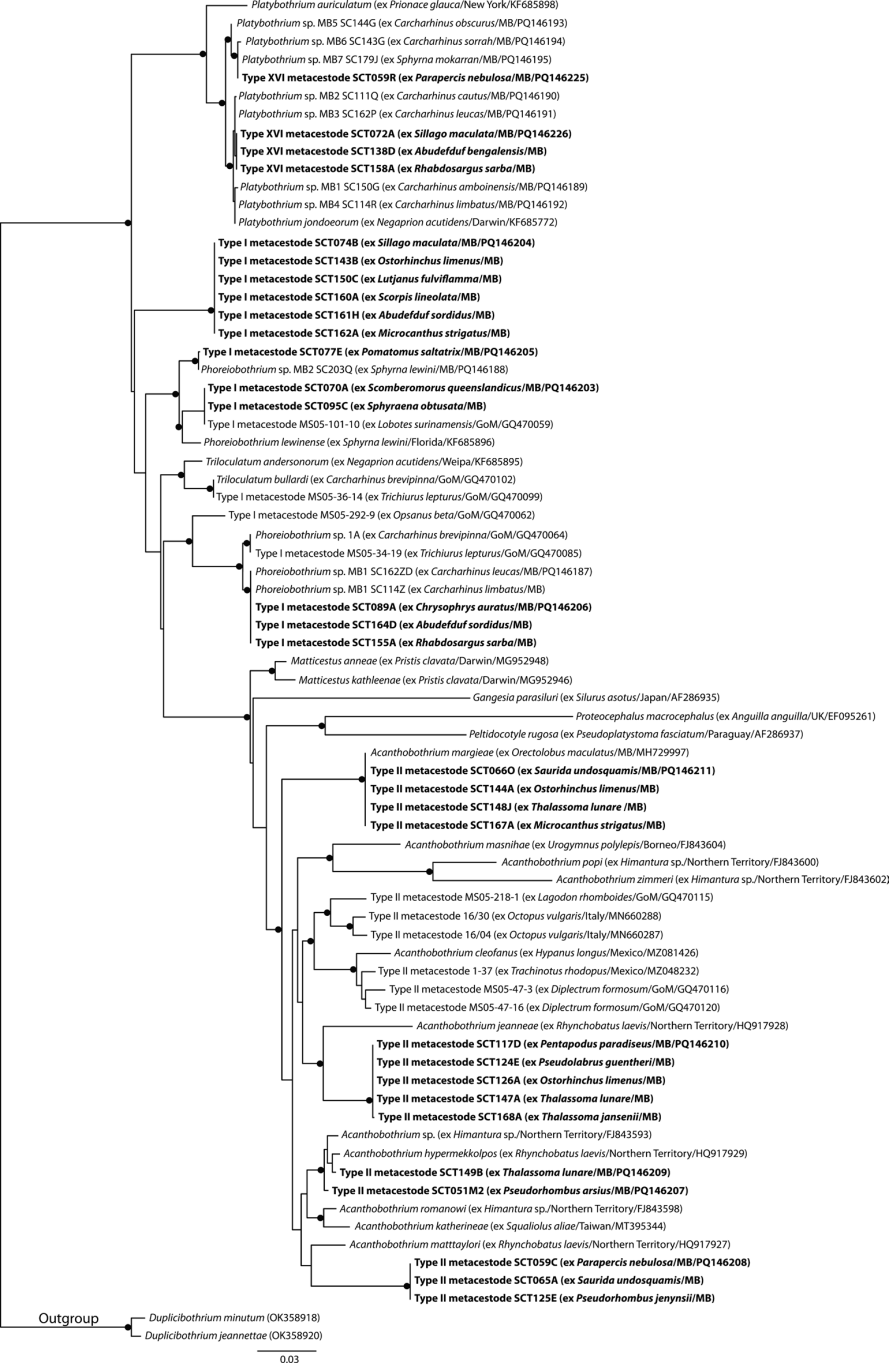
Phylogenetic tree from the Maximum likelihood analysis of the Onchoproteocephalidea dataset, incorporating Types I, II and XVI metacestodes. Strongly supported nodes (>80) are indicated by a filled circle. The scale-bar indicates expected number of substitutions per site. Abbreviations: GoM, Gulf of Mexico; MB, Moreton Bay.

Types I, II and XVI metacestodes were identified as onchoproteocephalideans. Based on the topology of Caira et al. (2020), the Onchoproteocephalidea analyses incorporated published sequence data of Types I and II metacestodes from teleost fishes of the Gulf of Mexico, Type II metacestode sequences from an octopus from the Mediterranean, adult worms of relevant onchobothriid genera, *Matticestus* Caira, Jensen & Fyler, 2018, and a select few proteocephalid genera. Sequences of Type I metacestodes from Moreton Bay form four clades, three of which form well-supported clades with species of *Phoreiobothrium* (Fig. 2). The fourth clade of Type I metacestodes resolve as basal to all the included taxa excluding species of *Platybothrium*; however, nodal support for this position is poor, and notably species of *Phoreiobothrium* do not form monophyletic clade in the analysis. Sequences of Type II metacestodes from Moreton Bay form four clades, all of which resolve in a large well-supported clade of adult *Acanthobothrium* and Type II metacestodes from the Gulf of Mexico and the Mediterranean. Sequences of Type XVI metacestodes from Moreton Bay form two clades which both resolve in a well-supported clade with sequences of adult *Platybothrium*. Although Jensen & Bullard (2010) matched Type I and Type II metacestodes to adult genera in the Gulf of Mexico, they did not encounter Type XVI, which is here clearly identified as relating to *Platybothrium*.

**Fig. 3:**
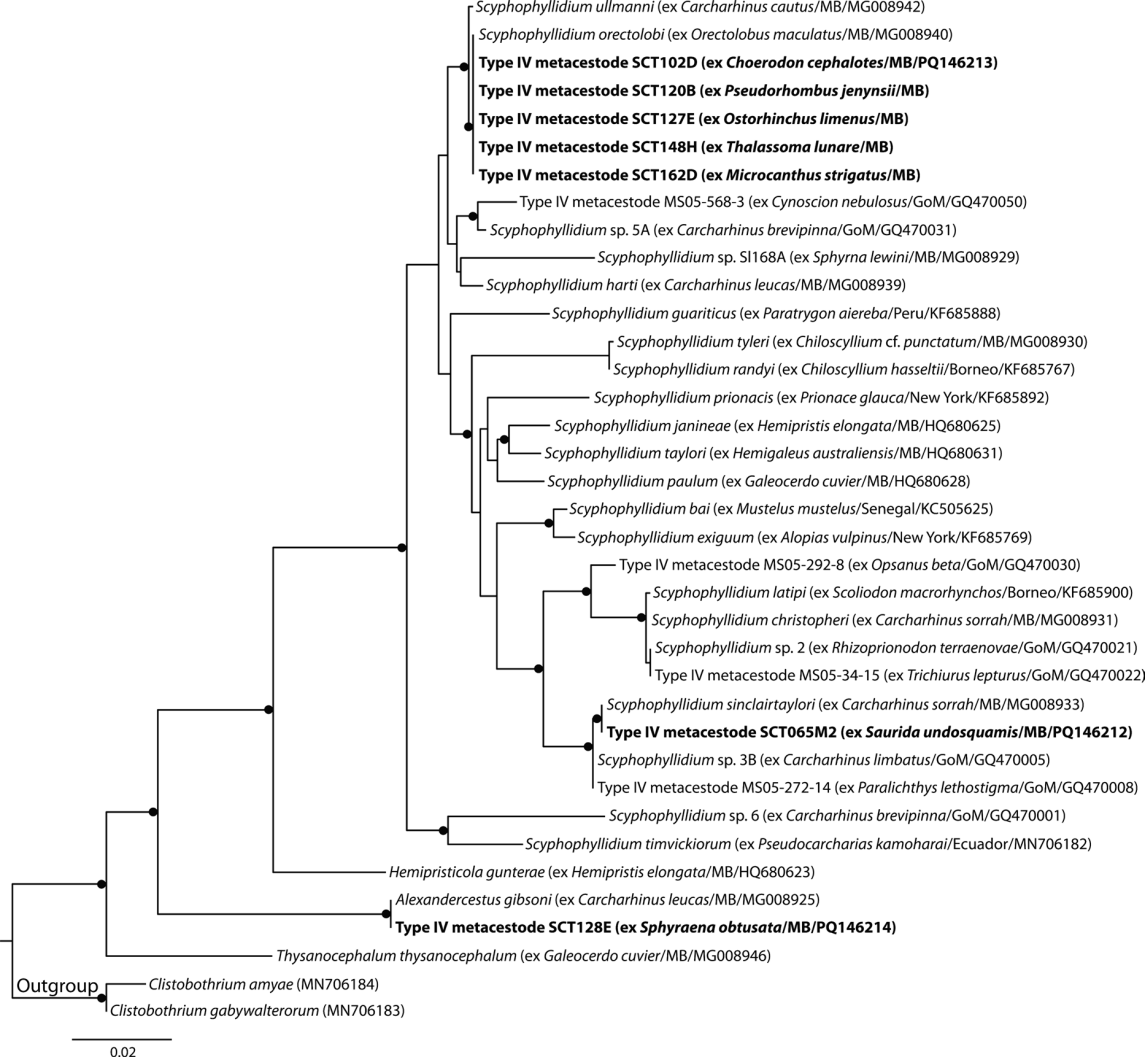
Phylogenetic tree from the Maximum likelihood analysis of the Phyllobothriidae dataset, incorporating Type IV metacestodes. Strongly supported nodes (>80) are indicated by a filled circle. The scale-bar indicates expected number of substitutions per site. Abbreviations: GoM, Gulf of Mexico; MB, Moreton Bay.

Type IV metacestodes were identified as phyllobothriids. Based on the topology of Caira et al. (2020), the Phyllobothriidae analysis incorporated sequence data for species of *Alexandercestus*, *Hemipristicola*, *Scyphophyllidium*, and *Thysanocephalum* from across the Pacific, Atlantic and Indian Oceans, and Type IV larval data from teleost fishes from the Gulf of Mexico. Sequences of Type IV metacestodes from Moreton Bay form three clades, all of which matched adult sequences, two species of *Scyphophyllidium* and one species of *Alexandercestus* (Fig. 3). Jensen & Bullard (2010) matched Type IV metacestodes to adult *Scyphophyllidium* (then *Paraorygmatobothrium*) in the Gulf of Mexico.

**Fig. 4:**
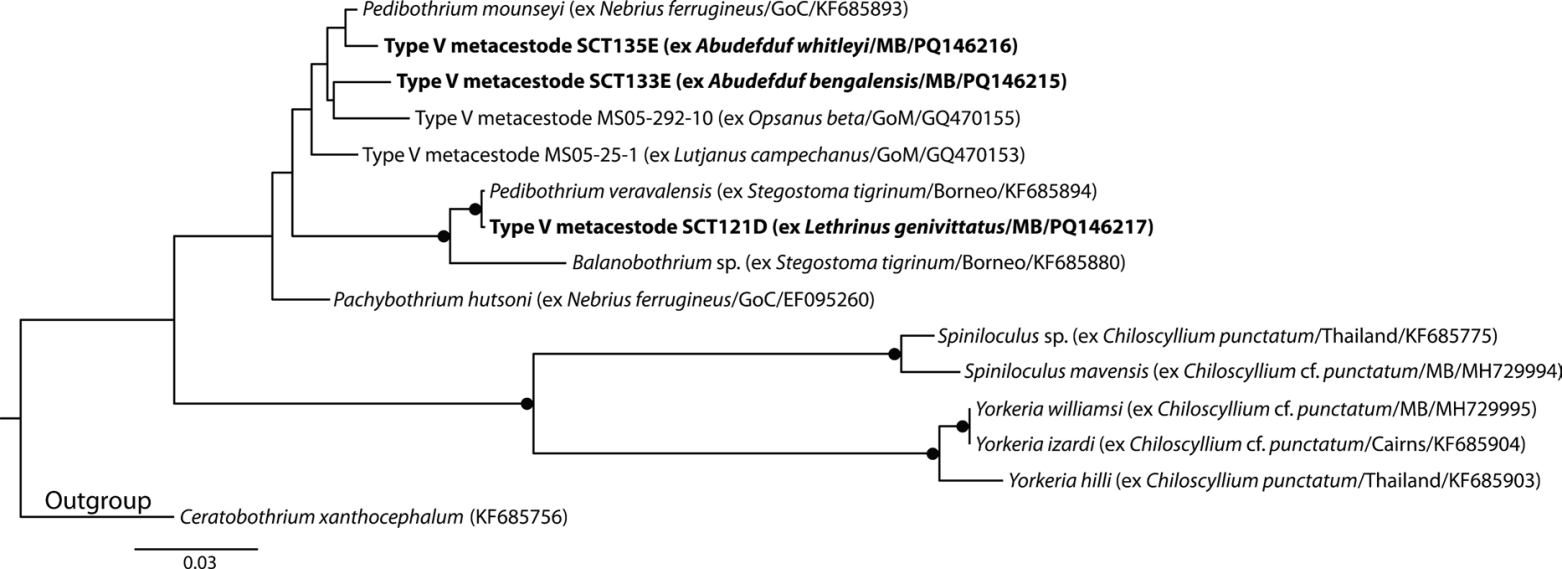
Phylogenetic tree from the Maximum likelihood analysis of the Balanobothriidae dataset, incorporating Type V metacestodes. Strongly supported nodes (>80) are indicated by a filled circle. The scale-bar indicates expected number of substitutions per site. Abbreviations: GoC, Gulf of California; GoM, Gulf of Mexico; MB, Moreton Bay.

Type V metacestodes were identified as balanobothriids. Based on the topology of Caira et al. (2017), the Balanobothriidae analysis incorporated sequence data of Type V metacestodes from teleost fishes from the Gulf of Mexico and adult *Balanobothrium* Hornell, 1911, *Pachybothrium*, *Pedibothrium*, *Spiniloculus* and *Yorkeria*. The sequences of Type V metacestodes from Moreton Bay represent three genotypes, each of which form a clade with sequences of adult *Pedibothrium* (Fig. 4); however, none of these larval sequences were an identical match to those of adult cestodes*.* Although Jensen & Bullard (2010) did not find an exact match for larval Type V, they inferred that it relates to species of *Pachybothrium* and/or *Pedibothrium*. It is clear from the current study that larval Type V relates to *Pedibothrium* and, given the close phylogenetic relationships of *Pedibothrium* and *Pachybothrium*, it seems plausible that this type might also represent *Pachybothrium*. Notably, *Pedibothrium* did not resolve as monophyletic, with a single sequence of an undescribed species of *Balanobothrium* rendering the genus paraphyletic.

**Fig. 5:**
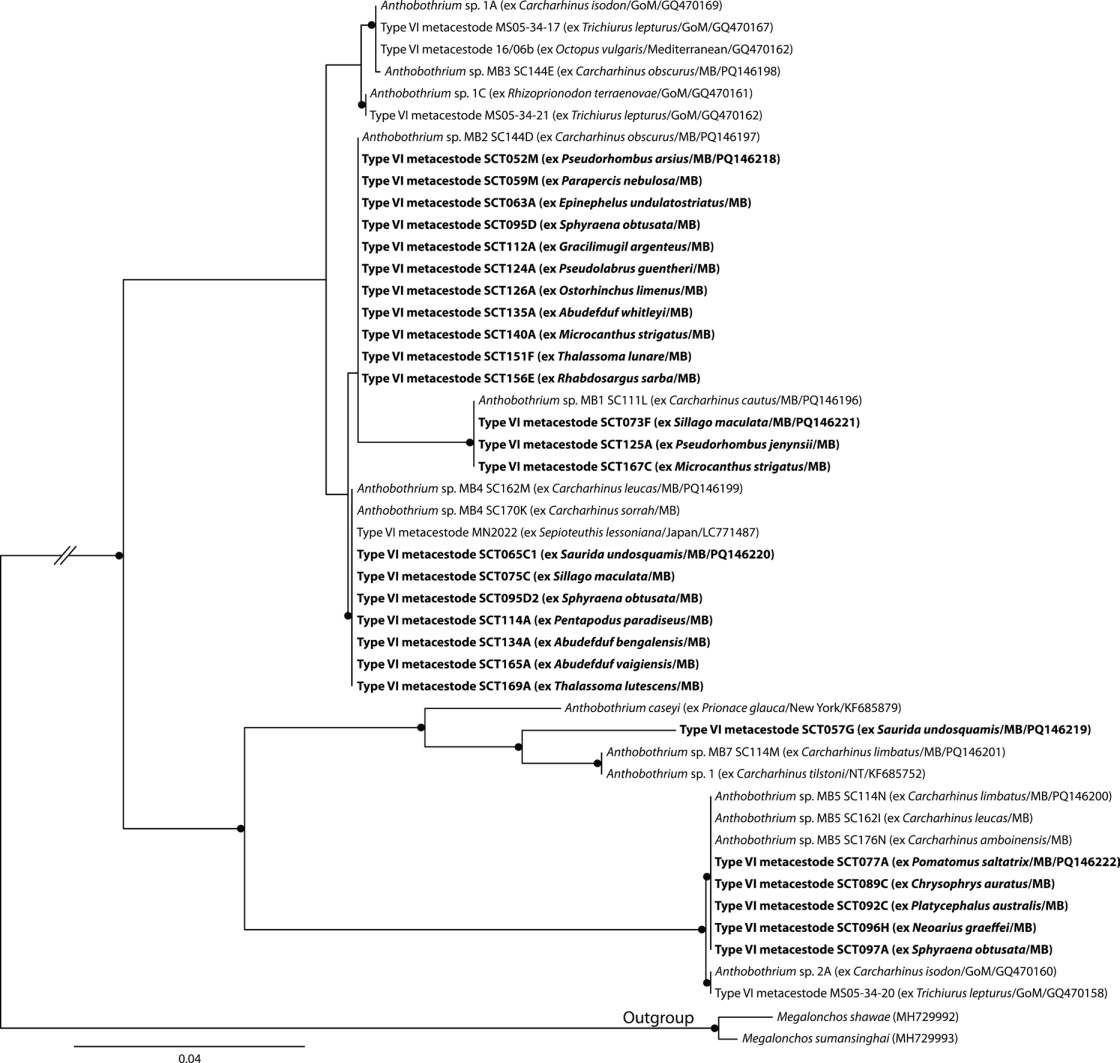
Phylogenetic tree from the Maximum likelihood analysis of the *Anthobothrium* dataset, incorporating Type VI metacestodes. Strongly supported nodes (>80) are indicated by a filled circle. The scale-bar indicates expected number of substitutions per site. Abbreviations: GoM, Gulf of Mexico; MB, Moreton Bay.

Type VI metacestodes were identified as *Anthobothrium* species. The analysis of these data incorporated sequences of Type VI metacestodes from teleost fishes United States waters, from a squid from Japanese waters and an octopus from the Mediterranean, and from adult *Anthobothrium* worms from carcharhinid sharks from Australia and the United States. Sequences of Type VI metacestodes from Moreton Bay form five clades, four of which match sequences of adult *Anthobothrium* specimens (Fig. 5). These findings support those of Jensen & Bullard (2010), who matched Type VI metacestodes to adult *Anthobothrium* in the Gulf of Mexico. Notably, based on results from *in vitro* cultivation, Chambers et al. (2000) also predicted that this larval type would represent species of *Anthobothrium*.

**Fig. 6:**
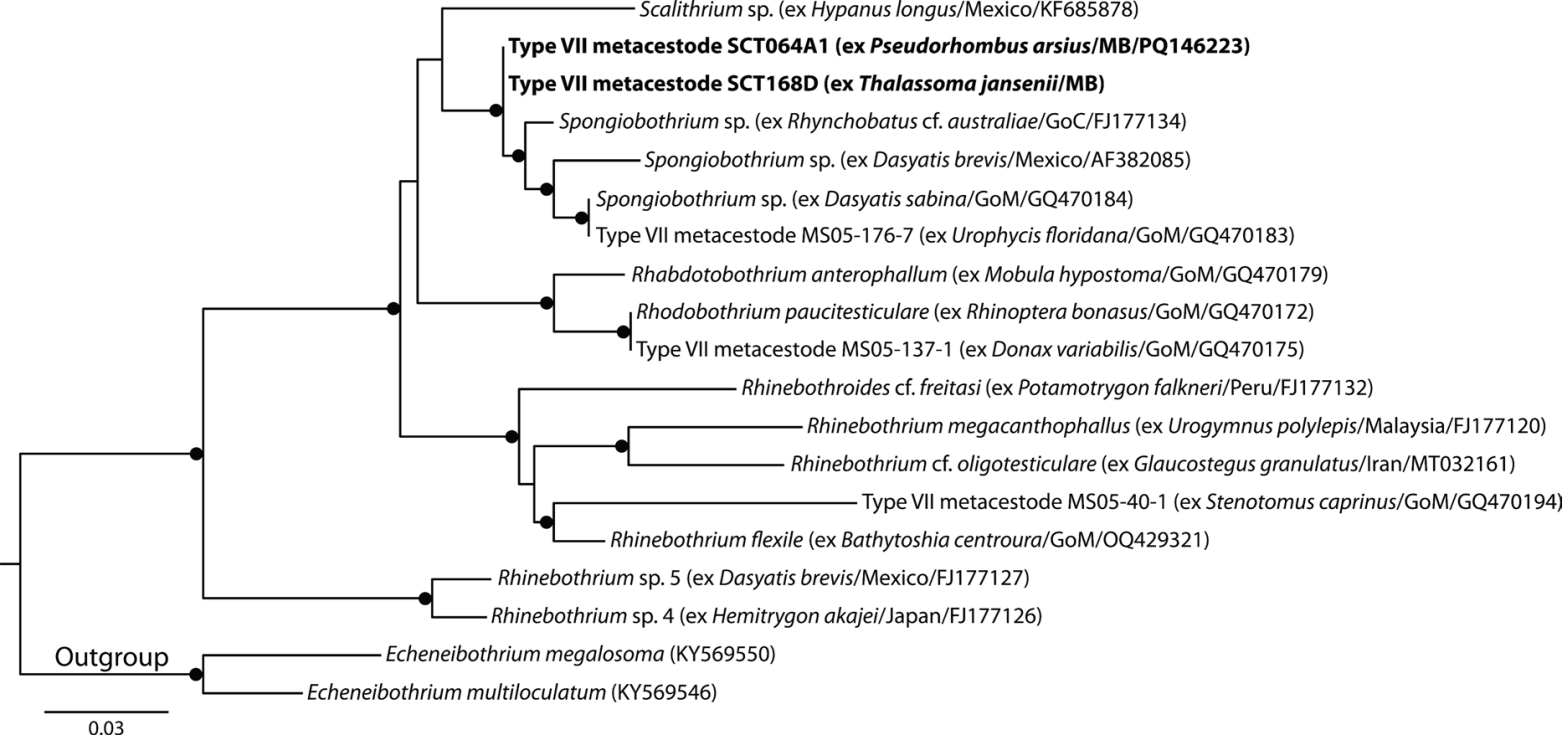
Phylogenetic tree from the Maximum likelihood analysis of the Rhinebothriidae dataset, incorporating Type VII metacestodes. Strongly supported nodes (>80) are indicated by a filled circle. The scale-bar indicates expected number of substitutions per site. Abbreviations: GoC, Gulf of California; GoM, Gulf of Mexico; MB, Moreton Bay.

Type VII metacestodes were identified as rhinebothiids. Based on the topology of Herzog et al. (2023), the Rhinebothriidae analysis incorporated sequences of Type VII metacestodes from fishes and bivalves of the Gulf of Mexico and of adult worms of the genera *Rhabdotobothrium* Euzet, 1953, *Rhinebothrium*, *Rhinebothroides* Mayes, Brooks & Thorson, 1981, *Rhodobothrium*, *Scalithrium* Ball, Neifar & Euzet, 2003 and *Spongiobothrium*. Sequences of Type VII metacestodes collected during this study form a clade with those of adult *Spongiobothrium* and Type VII metacestodes from the Gulf of Mexico (Fig. 6). Jensen & Bullard (2010) identified this larval type as relating to *Rhinebothrium* or *Spongiobothrium*.

**Fig. 7:**
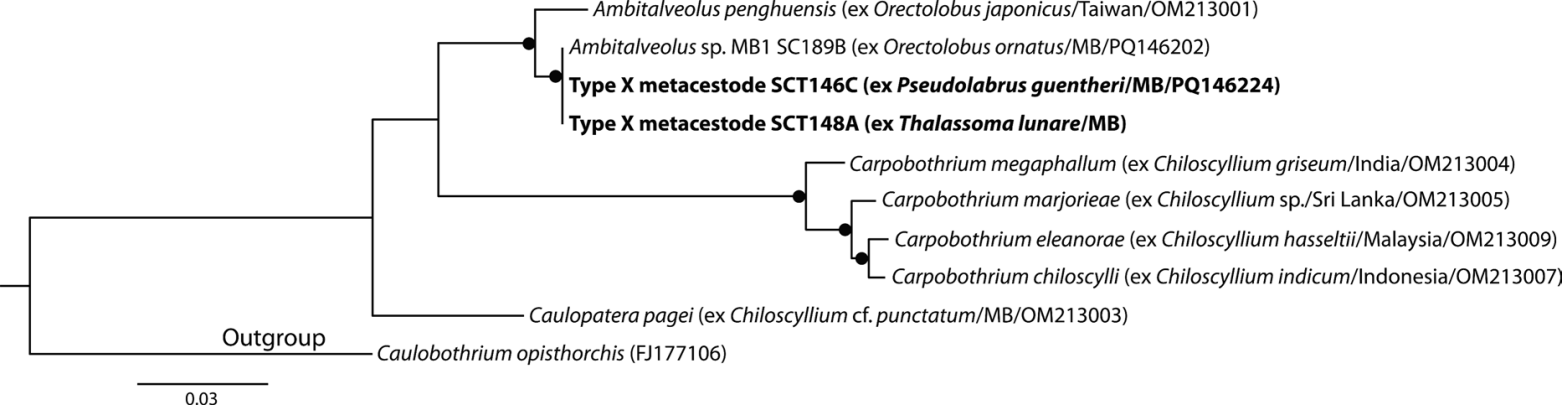
Phylogenetic tree from the Maximum likelihood analysis of the ‘tetraphyllidean Clade 3’ dataset, incorporating Type X metacestodes. Strongly supported nodes (>80) are indicated by a filled circle. The scale-bar indicates expected number of substitutions per site. Abbreviations: MB, Moreton Bay.

Type X metacestodes were identified as belonging to ‘tetraphyllidean Clade 3’ (*sensu* Caira et al., 2017). Based on the topology of Caira & Jensen (2022), the Clade 3 analysis incorporated sequences for adults of *Ambitalveolus*, *Carpobothrium* and *Caulopatera* from orectolobiform sharks of the tropical Indo-west Pacific. The sequences of Type X metacestodes represent a single genotype and match an undescribed species of *Ambitalveolus* collected from *Orectolobus ornatus* (De Vis), also from Moreton Bay (Fig. 7). This undescribed species differs from the only other *Ambitalveolus* for which sequence data are available, *Ambitalveolus penghuensis* Caira & Jensen, 2022 (also described form a species of *Orectolobus* Bonaparte), at 22 base positions. Jensen & Bullard (2010) speculated that Larval Type X related to *Carpobothrium*. While it is clear that the new specimens of this larval type relate to species of *Ambitalveolus*, it is plausible that all three genera in this clade will share the larval morphotype given the close phylogenetic relationship and similarities in bothridial structure.

### Metacestode types

**Larval Type I** *sensu* Jensen & Bullard (2010) (Fig. 1a)

*Generic identity*: *Phoreiobothrium* Linton, 1889 and *Triloculatum* Caira & Jensen, 2009 (Onchoproteocephalidea: Onchobothriidae).

*New hosts*: 21 species of Teleostei, see Table 1.

*Material deposited*: 20 voucher specimens (QM G 233050–69).

*Molecular sequence data*: D1-D3 region of the 28S rDNA gene, 13 sequences representing four genotypes (four sequences submitted to GenBank, PQ146203–06).

*Measurements*: Morphology consistent with diagnosis provided by Jensen & Bullard (2010). Body elongate, undivided, tapering posteriorly, length highly variable, 327–1,960 (905) long, 109–370 (217) wide. Apical sucker present, often funnel shaped, 47–141 (83) long, 58–130 (93) wide. Bothridia oval, 46–171 (115) long, 33–126 (81) wide, divided into two loculi; posterior loculus generally larger than anterior loculus.

*Remarks*: Notably, there was variation regarding body size and the shape of the apical sucker between specimens of this type; however, these differences did not align with genetic differences.

**Larval Type II** *sensu* Jensen & Bullard (2010) (Fig. 1b, c)

*Generic identity*: *Acanthobothrium* van Beneden, 1849 (Onchoproteocephalidea: Onchobothriidae).

*New hosts*: 11 species of Teleostei, see Table 1.

*Material deposited*: 48 voucher specimens (QM G233070–88, QM G233158–86).

*Molecular sequence data*: D1-D3 region of the 28S rDNA gene, 17 sequences representing five genotypes (five sequences submitted to GenBank, PQ146207–11).

*Measurements*: Morphology consistent with diagnosis provided by Jensen & Bullard (2010). Body elongate, undivided or divided, tapering posteriorly, length highly variable, 681–5,040 (1,527) long, 142–327 (219) wide. Apical sucker present, 42–100 (65) long, 51–119 (78) wide. Bothridia elongate, 101–254 (156) long, 58–139 (89) wide, divided into anterior pad and posterior loculus; posterior loculus usually subdivided by horizontal septa forming two to three loculi; posteriormost septa often inconspicuous.

*Remarks*: There was clear morphological variability within this type, with specimens of differing in the division of the bothridia (weak, inconspicuous septa *vs* strong, conspicuous septa), body length and the body divisions (undivided *vs* divided). Notably, all Type II metacestodes with some body division represent a single genotype, which is an identical molecular match to *Acanthobothrium margieae* Fyler, 2011.

**Larval Type IV** *sensu* Jensen & Bullard (2010) (Fig. 1d)

*Generic identity*: *Alexandercestus* Ruhnke & Workman, 2013 and *Scyphophyllidium* Woodland, 1927 (Phyllobothriidea: Phyllobothriidae).

*Hosts*: 16 species of Teleostei, see Table 1.

*Material deposited*: 20 voucher specimens (QM G 233089–108).

*Molecular sequence data*: D1-D3 region of the 28S rDNA gene, seven sequences representing three genotypes (three sequences submitted to GenBank, PQ146212–14).

*Measurements*: Morphology consistent with diagnosis provided by Jensen & Bullard (2010). Body tiny, elongate, undivided, tapering posteriorly, 192–351 (274) long, 78–137 (103) wide. Apical sucker present, 28–53 (38) long, 35–77 (51) wide. Bothridia circular to oval, undivided, 33–61 (47) long, 28–57 (40) wide.

**Larval Type V** *sensu* Jensen & Bullard (2010) (Fig. 1f)

*Generic identity*: *Pedibothrium* Linton, 1909 (Tetraphyllidea: Balanobothriidae).

*New hosts*: six species of Teleostei, see Table 1.

*Material deposited*: seven voucher specimens (QM G 233109–15).

*Molecular sequence data*: D1-D3 region of the 28S rDNA gene, three sequences representing three genotypes (three sequences submitted to GenBank, PQ146215–17).

*Measurements*: Morphology consistent with diagnosis provided by Jensen & Bullard (2010). Body tiny, elongate, undivided, tapering posteriorly, 276–334 (307) long, 120–174 (138) wide. Apical sucker absent. Bothridia oval, 74–114 (87) long, 46–77 (58) wide, divided into anterior and posterior loculi; division between loculi sometimes inconspicuous.

**Larval Type VI** *sensu* Jensen & Bullard (2010) (Fig. 1g)

*Generic identity*: *Anthobothrium* van Beneden, 1850 (Tetraphyllidea *incertae sedis*).

*New hosts*: 33 species of Teleostei, see Table 1.

*Material deposited*: 20 voucher specimens (QM G 233129–48).

*Molecular sequence data*: D1-D3 region of the 28S rDNA gene, 33 sequences representing five genotypes (five sequences submitted to GenBank, PQ146218–22).

*Measurements*: Morphology consistent with diagnosis provided by Jensen & Bullard (2010). Body elongate, undivided, tapering posteriorly, 518–1,009 (767) long, 147–292 (216) wide. Apical sucker present, 32–63 (51) long, 43–86 (64) wide. Bothridia roughly circular, undivided, 58–125 (94) long, 53–117 (86) wide.

**Larval Type VII** *sensu* Jensen & Bullard (2010) (Fig. 1h)

*Generic identity*: Likely *Spongiobothrium* Linton, 1889 (Rhinebothriidea: Rhinebothriidae).

*New hosts*: Labridae: *Thalassoma jansenii* (Bleeker), Jansen's wrasse. Paralichthyidae: *Pseudorhombus arsius* (Hamilton), Largetooth flounder.

*Material deposited*: 3 voucher specimens (QM G 233149–51).

*Molecular sequence data*: D1-D3 region of the 28S rDNA gene, three sequences representing one genotype (one sequence submitted to GenBank, PQ146223).

*Measurements*: Morphology consistent with diagnosis provided by Jensen & Bullard (2010). Body elongate, undivided, tapering posteriorly, 861–987 (929) long, 241–275 (257) wide. Apical sucker present, 102–108 (105) long, 106–125 (117) wide. Bothridia elongate, facially loculated, 174–235 (197) long, 106–134 (118) wide; loculation consisting of two columns of rectangular loculi.

**Larval Type X** *sensu* Jensen & Bullard (2010) (Fig. 1e)

*Generic identity*: *Ambitalveolus* Caira & Jensen, 2022 (Tetraphyllidea *incertae sedis*).

*New hosts*: Labridae: *Pseudolabrus guentheri* Bleeker, Günther's wrasse; *Thalassoma lunare* (Linnaeus), Moon wrasse.

*Material deposited*: Six voucher specimens (QM G 233152–57).

*Molecular sequence data*: D1-D3 region of the 28S rDNA gene, four sequences representing one genotype (one sequence submitted to GenBank, PQ146224).

*Measurements*: Morphology consistent with characterisation provided by Chambers et al. (2000) and Jensen & Bullard (2010). Body elongate, undivided, tapering slightly posteriorly, 1,819–3,536 (2,478) long, 484–578 (512) wide. Apical sucker present, 100 long (measurable for only one specimen), 95–140 (110) wide. Bothridia large, undivided, pouch-shaped, with muscular bands on anterior and posterior margins of aperture, 201–238 (217) long, 216–304 (258) wide.

**Larval Type XVI** (Fig. 1i)

*Generic identity*: *Platybothrium* Linton, 1890 (Onchoproteocephalidea: Onchobothriidae).

*New hosts*: 12 species of Teleostei, see Table 1.

*Material deposited*: 13 voucher specimens (QM G 233116–28).

*Molecular sequence data*: D1-D3 region of the 28S rDNA gene, four sequences representing two genotypes (two sequences submitted to GenBank, PQ146225–26).

*Measurements*: Body tiny, elongate, undivided, tapering posteriorly, 172–453 (314) long, 110–168 (128) wide. Apical sucker present, small, sometimes poorly defined. Bothridia oval, 66–114 (83) long, 49–75 (60) wide, divided into anterior and posterior loculi with division between loculi sometimes inconspicuous; anterior loculus smaller than posterior loculus.

*Remarks*: Type XVI metacestodes are highly morphologically similar to Type V metacestodes (*Pedibothrium*) but differ from this type in possessing a small (albeit sometimes inconspicuous) apical sucker.

## Discussion

The results of the present study both support and extend the unified morphological type system proposed by Jensen & Bullard (2010), in that almost all metacestodes collected from Moreton Bay were identifiable to one of the 15 types proposed in their study. However, the results of this study clearly demonstrate that morphological types will ultimately be best used to identify metacestodes to a group of related genera rather than any distinct genus. Type IV metacestodes were found to represent species of at least two genera (*Alexandercestus* and *Scyphophyllidium*) and the reliable topology of this clade permits the cautious prediction that this type likely also represents *Hemipristicola* (which resolves between *Alexandercestus* and *Scyphophyllidium*). While all Type I metacestodes matched species of *Phoreiobothrium*, based on the topology of the onchoproteocephalidean analysis it seems likely that, despite the poor nodal support, Type I metacestodes will also represent species of *Triloculatum*. Based on the current topology of the balanobothriid analyses, and topology of this group previously published (Caira et al., 2014), it seems likely that Type V metacestodes represent both *Pedibothrium* and *Balanobothrium* and may represent *Pachybothrium*. This similarity of morphology between closely related genera is not unexpected, however, given the simplified form of metacestodes. More rigorous examination of samples within these types (enabled by the incorporation of hologenophore specimens) may reveal distinctions between genera, but we suspect this level of distinction, and thus an expansion of the number of types, would not be advantageous for most researchers attempting identification of these cestode larvae.

The larval type of several tetraphyllidean genera prominent in Moreton Bay remains unknown, with no metacestodes matches found for *Caulopatera*, *Hemipristicola*, *Megalonchos*, *Spiniloculus*, *Thysanocephalum* or *Yorkeria*. Based on the findings that larvae of a single type can represent species of multiple genera, more extensive genetic sequencing of the current types may reveal matches for some of the unmatched adult genera from Moreton Bay. However, given the highly morphologically distinct adult forms of *Spiniloculus*, *Thysanocephalum* and *Yorkeria*, there are potentially multiple types still to be discovered in the region. The restriction of intermediate host collection to teleost fishes may explain the failure to find metacestodes of *Caulopatera*, *Spiniloculus* and *Yorkeria*, as species of these genera are almost exclusively reported from species of *Chiloscyllium* (Hemiscylliidae) (Caira et al., 2007a; Cutmore et al., 2010; Cutmore et al., 2018). The diet of *Chiloscyllium* species comprises primarily benthic invertebrates (Gauthier et al., 2019; Last & Stevens, 2009; Lowry & Motta, 2007) and it is likely that the intermediate stages for these three genera will be found there. Failure to identify the larval form of *Megalonchos* may be due to the distribution of the definitive host. *Megalonchos* species infect hemigaleid sharks of the genera *Chaenogaleus* Gill and *Hemipristis* Agassiz (Caira et al., 2007b), with both *Megalonchos* species sequenced from Moreton Bay collected from *Hemipristis elongata* (Klunzinger) (Cutmore et al., 2018). *Hemipristis elongata* is rarely found in Moreton Bay (Taylor & Bennett, 2013), and is more commonly encountered in tropical regions of Australia (Last & Stevens, 2009). The absence of a metacestode match for *Thysanocephalum thysanocephalum* (Linton, 1889) Braun, 1900 is more surprising, however, as *Galeocerdo cuvier* (Péron & Lesueur) is a partial piscivore and is considered common in the region (Johnson, 2010).

During this study we examined just 54 of 700+ teleost species known from Moreton Bay (Johnson, 2010). The number of individual fishes examined for most species were far too low to draw any decisive inferences on prevalence and richness of larvae. Despite the low dissection numbers, there were some notable patterns. Some fish species [e.g., *Microcanthus strigatus* (Cuvier), *Ostorhinchus limenus* Randall & Hoese, *Saurida undosquamis* (Richardson)] had both a high richness and high prevalence of metacestodes; all three of these species had 100% infection prevalence and were infected by five of the eight larval types. Some species [e.g., *Lethrinus genivittatus* Valenciennes and *Monodactylus argenteus* (Linnaeus)] had a high prevalence but low richness. And some species [e.g., *Atherinomorus vaigiensis* (Quoy & Gaimard) and *Gerres subfasciatus* Cuvier] were not infected at all, despite respectable number of examinations (12 and 13, respectively). While we do not think that all 700+ teleost species in Moreton Bay will harbour metacestodes, based on the findings above, it is clear that there are likely hundreds of individual host/parasite combinations yet to be identified.

Similar to prevalence and richness, the number of individual fishes examined for most species were far too low to allow definitive inferences on the host specificity of the larval types. However, of the eight larval types, seven were found in more than one order of fishes. Larval Types I, II, IV, and VI exhibit strikingly low host-specificity; Type VI metacestodes, which relate to species of *Anthobothrium*, were recovered from 21 families and 33 species of teleost fishes. There were five genotypes of Type VI larvae, of which four matched data for adult worms. Although the number of definitively identified intermediate hosts for each of these genotypes was relatively low (1–11 teleost species), even within these genotypes the host-specificity was clearly low. The four matched genotypes were recovered from three, five, six and 10 families of teleosts. In contrast, the Type X larvae appear to be highly host-specific, found only in two species of labrids (two of five *Pseudolabrus guentheri* and two of four* T*. *lunare* examined). An additional 15 teleost species were examined from the same location at which these infections were found, all of which were infected with several metacestode types but not by Type X.

In the only other broad survey of metacestodes infecting Australian fishes, Chambers et al. (2000) found a similar metacestode fauna, also identifying Types I, II, V, VI, VII, and X from a wide range of teleosts off Heron Island on the southern Great Barrier Reef. In a more taxonomically restricted study, Muñoz & Cribb (2005) and Muñoz et al. (2007) identified Types I, IV, V, VI, and VII from 14 species of labrids from Lizard Island, on the northern Great Barrier Reef. Several differences were observed between the present survey and those from the Great Barrier Reef. Chambers et al. (2000) did not collect any larval Type IV from teleosts off Heron Island; this absence is surprising as it is likely that several *Scyphophyllidium* species infect the many carcharhiniform and orectolobiform elasmobranchs found at Heron Island. Type IV larvae are by far the smallest metacestodes encountered in this study and it is possible that low infection levels were overlooked. However, Muñoz & Cribb (2005) identified what appears to be Type IV larvae from Lizard Island. One morphological type identified by Chambers et al. (2000) was absent from the present study; no specimens of larval Type IX (Chambers et al. Type 9) were collected from Moreton Bay. Muñoz & Cribb (2005) also identified several metacestode types not collected during this survey, identifying over 30 tetraphyllidean larval morphotypes from 14 species of labrids. There was no voucher material deposited by Muñoz et al. (2007) but based on the known fauna of elasmobranch hosts at Lizard Island, it seems unlikely that 30 genetically different larval types would be present at this location.

Notably, two larval types found during this study, Type X and Type XVI, were not collected by Jensen & Bullard (2010). Type X larvae were matched to *Ambitalveolus* and given the close phylogenetic relationship and similarities in bothridial structure, it seems plausible that this larval morphotype will also represent *Carpobothrium* and *Caulopatera*. All three of these genera have been reported from only orectolobiform sharks of the genera *Brachaelurus* (Brachaeluridae), *Chiloscyllium* (Hemiscylliidae), and *Orectolobus* (Orectolobidae). The absence of Type X in the Gulf of Mexico study by Jensen & Bullard (2010) is thus expected as species of *Brachaelurus*, *Chiloscyllium*, and *Orectolobus* are restricted to the Indo-west Pacific (Last & Stevens, 2009). The absence of Type XVI, identified as *Platybothrium*, in the collections of Jensen & Bullard (2010) is more perplexing, as there have been numerous *Platybothrium* species reported form sharks of the Atlantic (Healy, 2003), including from shark species examined as part of the Gulf of Mexico study. In contrast, Jensen & Bullard (2010) found two larval types, Type III and Type VIII, that were not collected from Moreton Bay. Jensen & Bullard (2010) identified Type III as *Duplicibothrium* and Type VIII as *Rhodobothrium*. The definitive hosts of these two genera, myliobatiform rays of the genera *Dasyatis*, *Myliobatis* and *Rhinoptera*, are abundant in Moreton Bay, and we suspect species of these genera will be found in the region. We predict that the absence in the current study is due to the restriction of our examinations to teleosts; Jensen & Bullard (2010) found both Type III and Type VIII in bivalve and gastropod hosts.

The low host-specificity of most of the larval types reported here is consistent with effective transmission to sharks that themselves have low dietary specificity. Carcharhiniform sharks identified as hosts of adult cestodes reported in this study have been widely shown to have exceptionally low dietary specificity (e.g., Salini et al., 1992; Simpfendorfer et al., 2001; Tillett et al., 2014; White et al., 2004). Dietary ranges of some orectolobiforms are similarly broad, with Huveneers et al. (2007) identifying five orders of teleosts, three orders of elasmobranchs and one order of cephalopod in the diet of the *Orectolobus maculatus* and six orders of teleosts and two orders of cephalopods in the diet of the *O*. *ornatus*. Evidently, there is typically no evolutionary pressure or advantage in cestode specialisation for a more restricted range of intermediate hosts than the dietary range of the definitive host. Overall, it is noteworthy that the generally high prevalence and low host-specificity of tetraphyllidean metacestodes in teleost fishes implies that transmission success of metacestodes is quantitatively very low.

## Data Availability

The data that support the findings of this study are available from the corresponding author upon reasonable request.
